# Supplementary material for: Apicortin, a Constituent of Apicomplexan Conoid/Apical Complex and Its Tentative Role in Pathogen—Host Interaction
Source: Trop Med Infect Dis. 2021 Jun 30;6(3):118. doi: 10.3390/tropicalmed6030118 (PMC8293464; doi:10.3390/tropicalmed6030118)
Supplement: Supplementary file 1 [file tropicalmed-06-00118-s001.zip › tropicalmed-1262391-supplementary.pdf]

Tropical Medicine and Infectious Disease

MDPI

Supplementary material of Apicortin, a Constituent of Apicomplexan Conoid/Apical Complex and Its Tentative Role in Pathogen–Host Interaction

Table S1. Apicortin in apicomplexan species.

Class/Subclass	Order/Suborder	Family	Genus	Species	Accesion Number
Aconoidasida	Haemosporida	Haemoproteidae	*Haemoproteus*	*H. tartakovskyi*	GGWD01002623[a]
		Plasmodiidae	*Hepatocystis*	*Hepatocystis sp.*	VWU48825
			Plasmodium	*P. falciparum*	XP_002808695
				P. reichenowi	SOV76822
				P. gaboni	XP_018643113
				P. sp. gorilla clade	XP_028537022
				P. sp. DRC-Itaito	SPJ09023
				P. coatneyi	XP_019915329
				P. brasilianum	MKLA02000337[b,c]
				P. cynomolgi	XP_004222837
				P. fragile	XP_012336263
				P. gonderi	XP_028543969
				P. inui	XP_008815147
				P. knowlesi	CAA9988661
				P. malariae	XP_028862044
				P. ovale	SCQ16487
				P. vivax	VUZ96467
				P. berghei	XP_034423003
				P. chabaudi	XP_016654315
				P. vinckei	XP_008623567
				P. yoelii	XP_729403
				P. gallinaceum	XP_028530755
				P. relictum	XP_028533490
	Nephromycida		*Cardiosporidium*	*C. cionae*	KAF8822549
			Nephromyces	*Nephromyxes sp.*	GHIL01164954[a]
	Piroplasmida	Babesiidae	*Babesia*	*B. bigemina*	XP_012767358
				B. bovis	XP_001609847
				B. divergens	CCLB01000001[b]
				B. microti	-
				B. ovata	XP_028867856
				Babesia sp.	XP_028871112
		Theileridae	*Theileria*	*T. annulata*	XP_952938

				T. equi	XP_004830792
				T. orientalis	PVC53896
				T. parva	EAN31621
Conoidasida/ Coccidia	Agamococcidiorida	Rhytidocystidae	*Rhytidocystis*	*Rhytidocystis sp.* [f]	GHVS01057697 [a,e]
					GHVS01047420 [a,e]
	Eucoccidiorida/ Eimeriorina	Cryptosporidiidae	*Cryptosporidium*	*C. andersoni*	OII78232
				C. baileyi	JIBL01000123[b]
				C. bovis	VHIT01000034[b]
				C. cuniculus	PVQC01000007[b]
				C. felis	KAF7458849
				C. hominis	OLQ16283
				C. meleagridis	POM85076
				C. muris	OLQ16283
				C. parvum	XP_001388280
				C. ryanae	VHLK01000036[b]
				C. sp. chipmunk	JXRN01000030[b]
				C. sp. 37763	SCFC01000030[b]
				C. tyzzeri	TRY51485
				C. ubiquitum	XP_028876475
				C. viatorum	QZWW01000032[b]
		Eimeriidae	*Cyclospora*	*C. cayetanensis*	XP_026194446
			Eimeria	*E. acervulina*	XP_013250787
				E. brunetti	CDJ52911
				E. falciformis	NPHX01000419[b]
				E. maxima	CBUY010002544[b]
					CBUY010001829[b]
				E. mitis	XP_013356320
				E. necatris	XP_013434470
				E. nieschulzi	JRZD01000823[b]
				E. praecox	CBUU010030987[b]
					CBUU010036236[b]
				E. tenella	XP_013231224.
		Sarcocystidae	*Besnoitia*	*B. besnoiti*	XP_029217437
			Cystoisospora	*C. suis*	PHJ23653
			Hammondia	*H. hammondi*	XP_008888750[d]
			Neospora	*N. caninum*	XP_003883150
			Sarcocystis	*S. neurona*	JAQE01000498[b]
			Toxoplasma	*T. gondii*	XP_002364910

Conoidasida/ Gregarinasina	Archigregarinorida	Selenidiidae	*Selenidium*	*S. pygospionis*	GHVN01000425[a,e]
			Digyalum	*D. oweni* [g]	GHVM01057186[a,e]
	Eugregarinorida	Cephaloidophoridae	*Cephaloidophora*	*C. cf. communis*	GHVH01004777[a,e]
		Gregarinidae	*Gregarina*	*G. niphandrodes*	XP_011128898
		Lecudinidae	*Ascogregarina*	*A. taiwanensis*	ABJQ01000568[b]
			Lankesteria	*L. abbotti*	HBHB01002866[a,e]
Conoidasida	Protococcidiorida	Eleutheroschizonidae	*Eleutheroschizon*	*E. duboscqi*	GHVT01063535[a]

The complete genomes of the species listed here have been sequenced (cf. https://www.ncbi.nlm.nih.gov/genome) except those of the species with grey background. Accession numbers are from the NCBI protein and nucleotide databases. Accession numbers with red background indicate that the sequence is incorrect in the NCBI protein database. Corrected sequences can be found in reference [1]. [a] –TSA (Transcriptome Shotgun Assembly); [b] –WGS (Whole Genome Shotgun); [c] – 100% identical with *P. malariae* XP_028862044; [d] – This record was removed. WGS: AVCM01036308; [e] – incomplete sequence; [f] – According to the new classification described in reference [2], *Rhytidocystis sp.* belongs to a new class 'Marosporida'; [g] – According to the new classification described in reference [2], *Digyalum oweni* belongs to the class 'Squirmidea', which does not belong to Apicomplexa.

References

1. Orosz, F. Wider than thought phylogenetic occurrence of apicortin, a characteristic protein of apicomplexan parasites. *J. Mol. Evol.* **2016**, *82*, 303–314, doi:10.1007/s00239-016-9749-5.
2. Mathur, V.; Kwong, W.K.; Husnik, F.; Irwin, N.A.T.; Kristmundsson, Á.; Gestal, C.; Freeman, M.; Keeling, P.J. Phylogenomics identifies a new major subgroup of apicomplexans, Marosporida *class nov*., with extreme apicoplast genome reduction. *Genome Biol. Evol*. **2021**, *13*, evaa244, doi:10.1093/gbe/evaa244.
